# A Giant Pediatric Chylolymphatic Cyst: An Extremely Rare Entity

**DOI:** 10.1097/PG9.0000000000000274

**Published:** 2022-12-08

**Authors:** Roopali Sehrawat, Nalini Bansal, Harshita Kour, Anand Sinha

**Affiliations:** From the * Department of Pathology, SGT University, Gurugram, Haryana; † Department of Paediatrics Surgery, Fortis Memorial Research Institute, Gurugram, Haryana.

**Keywords:** chylolymphatic cyst, mesenteric cyst, pediatric chylolymphatic cyst

## Abstract

Chylolymphatic cyst, variant of mesenteric cyst, is a rare entity. Clinical presentation and radiological features are not characteristic, so diagnosis can be made finally on histopathology. We report an extremely rare case of giant chylolymphatic cyst measuring >15 cm. A 2-year-old female presented with abdominal pain and vomiting. On examination, a ill-defined and firm mass was palpable just below umbilicus. Positron emission tomography-computed tomography scan revealed a large ill-defined lesion, which measured 16 × 13.2 × 6.7 cm in size and was seen in relation to the abdominal mesentery. Provisional diagnosis of mesenteric cyst was made. Laparotomy revealed multiple lymphatic cysts of variable size arising from the mesentery of proximal ileum. Histopathology examination confirmed the presence of a giant chylolymphatic cyst. Chylolymphatic cysts are rare entity and should be kept in mind while diagnosing a pediatric case of abdominal cysts.

## INTRODUCTION

Mesenteric cysts are a rare entity and chylolymphatic cyst, a variant of mesenteric cyst is even more rare. Literature regarding chylolymphatic cyst presenting in adult population is frequently available but not so in pediatric population. Moreover, clinical presentation and radiological features are not characteristic, so through our case report and literature review, we compiled the usual presentation, morphological features, and management of these cases for better and accurate diagnosis in future. We herein report an extremely unusual case of giant chylolymphatic cyst involving mesentery in a 2-year-old female child.

## CASE REPORT

**FIGURE 1.**
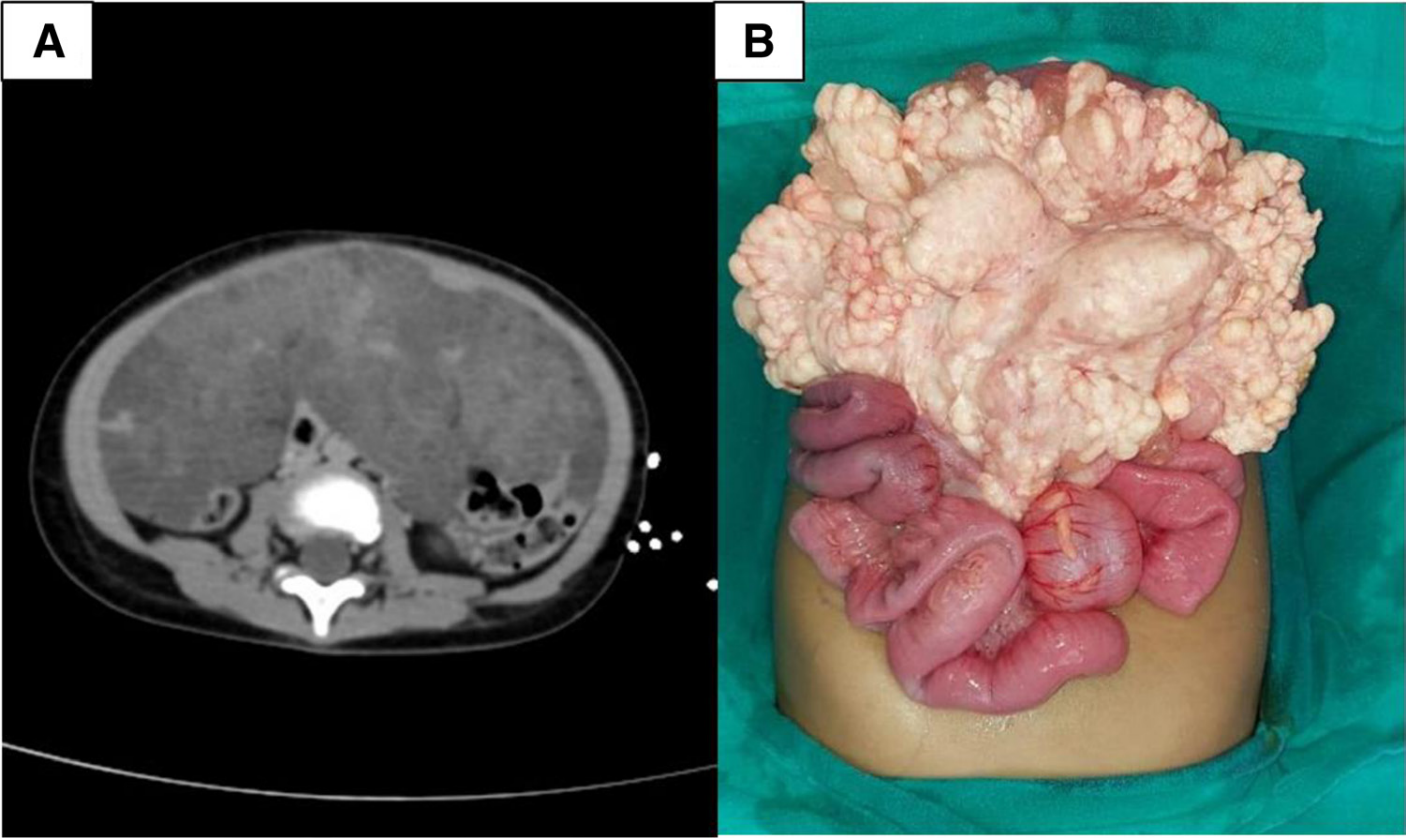
Positron emission tomography-computed tomography scan image and intraoperative image. A) Positron emission tomography-computed tomography scan image showing large ill-defined mass measuring 16 × 13.2 × 6.7 cm in size seen in relation to the abdominal mesentery and involving umbilical, infra umbilical, and hypogastric region with extension into the bilateral ileal fossa. B) Intraoperative image of multiloculated cyst of variable size arising from mesentery.

**FIGURE 2:**
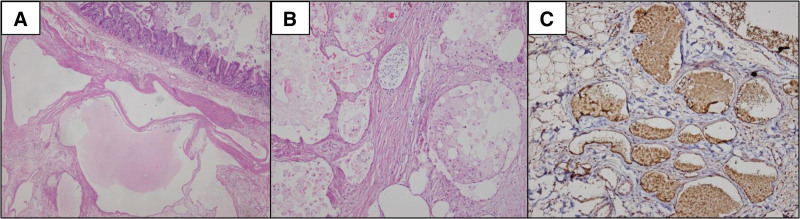
Microscopic features of multiloculated cyst. A) Ill-defined and variable size cystic spaces with overlying intestinal mucosa. (hematoxylin and eosin stain, ×10). B) Cystic spaces are lined by flattened endothelial cells and filled with foamy histiocytes and proteinaceous fluid (H&E, ×40). C) Immunohistochemistry, D2–40 positive in lymphatic endothelial lining (×40).

We report a case of 2-year-old female child who presented with complaints of pain in abdomen for 3 days and 2 episodes of nonbilious vomiting. On examination, vitals were stable. A vague mass was palpable just below umbilicus with ill-defined margins and firm surface. A Ultrasonography whole abdomen was performed, and there was evidence of multicystic intra-abdominal lesion. Positron emission tomography-computed tomography scan revealed a large ill-defined infiltrative mass lesion seen in relation to the abdominal mesentery, centered within the mesocolonic fat. The lesion measured 16 × 13.2 × 6.7 cm in size involving almost the entire abdominal cavity and demonstrate no significant contrast enhancement (Fig. 1A). The lesion was seen involving the umbilical, infra umbilical, and hypogastric region with extension into the bilateral ileal fossa. Provisional diagnosis of mesenteric cyst was made. Laparotomy revealed multiple lymphatic cysts of variable sizes arising from the mesentery of proximal ileum (25 cm proximal to ileocecal junction). Cysts were closely related to the intestine on the mesenteric surface and extending up to the root of mesentery. Approximately 25 cm of ileum was resected along with the mass and anastomosis was performed (Fig. 1B). Gross examination revealed a large multicystic mass with variable small size cysts measuring 17 × 10 × 4 cm in size. On serial sectioning, milky white fluid came out. On microscopy, there were variable sized and anastomosing ill-defined cystic spaces with thin walls. The cysts were lined by flatted endothelial cells with many cystic spaces, which were filled with foamy histiocytes and proteinaceous fluid (Fig. 2A, B) On Immunohistochemistry, D2–40 highlighted the lymphatic endothelial lining (Fig. 2C) Based on histology and immunohistochemistry studies final diagnosis of chylolymphatic cysts was rendered. Patient is doing well on 6 months of follow-up.

## DISCUSSION

A chylolymphatic cyst is a rare variant of a mesenteric cyst with a prevalence of 1 per 20 000 children (1). A chylolymphatic cyst, as the name suggests, is a cyst that contains both chyle and lymph, usually presents within the mesentery lined with a thin endothelium or mesothelium (1,2).

These cysts are thought to originate from isolated or ectopic lymphatic channels due to accumulation of both chyle and lymph in the small bowel mesentery. The accumulation in these channels results from an imbalance between the inflow and outflow of fluid (3).

Chylolymphatic cysts are diagnosed by 10 years of age in 25% of cases (2). Our case presented at the age of 2 years. In the evaluated series, age ranged from 2 months to 16 years.

Chylolymphatic cyst has varied presentation, it may be asymptomatic and may present with abdominal distension or may be associated with complications such as intestinal obstruction, volvulus, infection, rupture of the cyst, or obstruction of the urinary or biliary tract (1).

In most of these cases, prior diagnosis of the chylolymphatic cyst was difficult as no characteristic features were present on radiographs. Abdominal radiographs usually reveal air fluid levels in dilated loops of bowel only when associated with intestinal obstruction caused by compression of the adjacent bowel from the enlarged cyst or may be caused by mesenteric volvulus. Fujita et al (4) described fluid-fluid levels on abdominal ultrasound resulting from lighter chyle representing upper levels over a heavier lymph representing lower half (5). Computed tomography also provides similar findings based on the echo densities of different fluid levels described by some authors (1).

Most cases of the chylolymphatic cyst are managed by surgical excision of cyst with or without bowel resection. Others include enucleation or drainage of cyst. Lee et al (6) suggested that whenever possible conservative management such as partial cyst excision can be attempted without resulting in any recurrence.

Histopathology is important as it is not only confirmatory but also helps to exclude other differentials such as cystic lymphangioma, lymphoepithelial cyst, and multilocular peritoneal inclusion cysts. Cystic lymphangioma has distinct histology, the smooth muscle and lymphatic spaces are lacking in the wall of cyst. Some literature has considered chylolymphatic cysts to be a variant of mesenteric cysts, while other considered it as a type of cystic lymphangioma. Multilocular peritoneal inclusion cyst is differentiated from chylolymphatic cyst by presence of mesothelial cells (1,7). Differential diagnosis of chylolymphatic cyst on histology is discussed in Table 1.

**TABLE 1.** Differential diagnosis of chylolymphatic cyst

Features	Chylolymphatic cyst	Cystic lymphangioma	Multilocular peritoneal inclusion cyst	Lymphoepithelial cysts
Gross appearance	Unilocular/multilocular cyst	Unilocular/multilocular	Multilocular cyst	Unilocular/multilocular
Cyst lining	Endothelial lining	Endothelial lining	Mesothelial lining	Stratified squamous lining which may be denuded at places
Cyst content	Milky fluid	Clear, serous, chylous, or hemorrhagic fluid	Yellow and watery to gelatinous or serosanguineous or bloody	The cyst contents may vary from serous to cheesy/caseous-appearing depending on the degree of keratin formation
Microscopy	Lymphoid tissue and foam cell	Lymphoid tissue and foam cell	Clear fluid	Lymphoid aggregates in wall of cyst
Immunohistochemistry	D2–40	D2–40	Calretinin and Wilms tumor 1	High molecular weight cytokeratin

## GIANT PEDIATRIC CHYLOLYMPHATIC CYST

On extensive search on PubMed with keywords “paediatric chylolymphatic cyst” only 12 articles were found representative of 17 patients (Table 2) (1,2,5,7–14).

**TABLE 2.** Characteristics of pediatric patients with chylolymphatic cyst reported in the review

Journal name	Author	Size of cyst	Salient clinical features	Treatment	Follow-up
*Afr J Paediatr Surg*.	Tripathy et al (8) 2022	—	2-month-old female infant	Cyst excision along with adjoining ileum	No evidence of recurrence
*Radiol Case Rep*.	Pai et al (3) 2021	13.5 cm cystic lesion	16-year-old female presented with acute abdominal pain, vomiting, and severe back pain	En bloc cyst removal with ligation of the lymphatic feeder vessels	No evidence of recurrence
*Folia Med Cracov*.	Patoulias et al (9) 2020	—	3-month-old male infant presented with complaint of repeated vomiting of undigested material	Cyst along with adjacent intestinal helix was resected followed by an end-to-end intestinal anastomosis in 2 layers	No follow-up
*Turk J Surg*.	Mohamed (7) 2018	9.4 cm × 12.2 cm size cyst	1-year old presented with gradual distension of the abdomen for 4 months with occasional episodes of vomiting and a lobulated cystic abdominal mass on palpation	Surgical excision of all cysts was done in toto	No follow-up
*J Clin Diagn Res*.	Ghritlaharey and Morel (10) 2014	10 × 8 cm size cyst	8-year-old boy presented with abdominal pain, vomiting, abdominal distension, and constipation (features of acute intestinal obstruction) of 5 days duration	Cyst excision along with part of ileum was done en bloc. An ileostomy was created due to gross disparity in the lumen of the ileum, which was closed 2 and half month later	No follow-up
*Indian J Urol*.	Meitei et al (11) 2013	—	A 5-year-old male child presented with left scrotal swelling and abdominal distension	Excision of cyst along with adjacent omentum. Internal inguinal ring was closed with a purse string suture	No follow-up
*J Med Case Rep*.	Rattan et al (1) 2009	Varying sized cyst with the smallest approximately 8 mm in diameter and the largest approximately 9 cm in diameter	8 patients were in the age range 18 months to 10 years. Of these 8 patients, 4 presented with an abdominal lump and 2 each with abdominal pain and acute intestinal obstruction	Complete cyst excision along with involved gut was done in all cases	No recurrence during follow-up period which ranged from 4 months to 8 years
*Surg Today*.	Ratan et al (12) 2003	15 × 15 cm size cyst	4-year-old boy presented with an acute intestinal obstruction	Cyst excision along with part of bowel involved	No recurrence occurred after 1 years of follow-up.
*Indian J Pediatr*.	Panjwani et al (13) 1993	20 × 15 × 10 cm size cyst	10 days old neonate presented with complaint of bilious vomiting and refusal of feeds. On examination, the abdomen was grossly distended	Cyst excision along with resection of jejunum (10 cm) and end-to-end anastomosis done	No follow-up
*J Indian Med Assoc*.	Choudhury et al (14) 1976	—	—	—	—
—, Data not available.					

Chylolymphatic cyst of size >15 cm are considered as giant chylolymphatic cyst. Of reviewed cases of pediatric chylolymphatic cysts, only 2 are giant chylolymphatic cysts. In the evaluated series of pediatric chylolymphatic cyst most common presentation was with abdominal pain and other symptoms include vomiting, abdominal distension, constipation, acute intestinal obstruction, severe back pain, and scrotal swelling. Our case presented with complaints of pain in abdomen and vomiting.

On follow-up of our case, there was no recurrence. In the evaluated series, there was no recurrence in any of the case except in 5 cases where no follow-up was done.

## CONCLUSIONS

Chylolymphatic cysts area rare entity and should be kept in mind while diagnosing a pediatric case of abdominal cysts. In evaluated series, the most common presentation was abdomen distention and abdominal pain. Treatment involves excision of cyst with or without resection of bowel. Diagnosis is usually made on histopathological examination.
